# Short and long arm cast and pain after discharge in children who underwent reduction of distal forearm fracture in the Emergency Department: A study protocol for a randomized comparative effectiveness study

**DOI:** 10.1016/j.conctc.2018.06.003

**Published:** 2018-06-02

**Authors:** Martina Giacalone, Tali Capua, Itai Shavit

**Affiliations:** a Department of Paediatric Medicine, Anna Meyer Children's University Hospital, Florence, Italy; b Emergency Department, Dana-Dwek Children's Hospital, Tel Aviv Sourasky Medical Center, Tel Aviv, Israel; c Emergency Department, Ruth Children's Hospital, Rambam Health Care Campus, Haifa, Israel

**Keywords:** Pain, Children, Fracture, Cast, ED, emergency department, DFF, distal forearm fracture

## Abstract

Distal forearm fracture is the most common fracture in childhood. Patients with this type of injury suffer from meaningful pain after Emergency Department (ED) discharge. Previous studies demonstrated that short arm (below-the-elbow) casts perform as well as long arm (above-the-elbow) casts for maintaining the reduction of distal forearm fractures, with a similar rate of complications. Consequently, short casts are the commonly used method of immobilization after closed reduction of a distal forearm fractures in children older than 4 years. However, short casts carry a potential disadvantage; since they cannot prevent supination in a wrist that is held in pronation, and vice versa, their use might be associated with pain.

We initiated this study to examine the effect of the type of casting on post discharge pain. We will conduct an open-label randomized, controlled trial comparing short cast immobilization with long cast immobilization in children who had a reduction of distal forearm fracture in the ED. Our hypothesis is that children with distal forearm fractures who are treated with a long cast, experience less pain during the first 48 h after ED discharge than children who are treated with a short cast.

## 1 Introduction

Distal forearm fractures (DFF) are the most common fractures in childhood. Most of these injuries are successfully treated non-operatively with closed reduction under procedural sedation and cast immobilization. This conservative technique is possible because these fractures heal rapidly and have potential for remodeling [[1], [2], [3], [4]]. Previous studies indicate that short arm (below-the-elbow) casts perform in children as well as long arm (above-the-elbow) casts for maintaining the reduction of DFFs, with a similar rate of complications [[5], [6], [7], [8], [9], [10]]. Contrary to the fracture-care principle of immobilizing the joint proximal to and distal to a fracture, it appears that the immobilization of the long cast offers no benefit in maintaining the alignment of these fractures. This may be because the elbow joint is distant from the fracture, and the majority of immobilization is secured over the length of the forearm. Previous studies show that children treated with a long cast missed significantly more days of school and were more likely to require assistance with various activities of daily living activities compared to children who were treated with a short cast [6,9]. Consequently, the commonly used method of immobilization after closed reduction of DFF is a short cast. In children under the age of 4 years, however, a long cast is still recommended, as short arm casts may slip [11].

However, short arm casts, cannot successfully prevent supination in a wrist that is held in pronation, and vice versa [12]. Supination and pronation have been associated with pain in children with DFF. Therefore, it is reasonable to assume that a long cast would be associated with less pain after fracturing a distal forearm [13,14].

A previous study that analyzed pain after reduction and casting of different types of upper extremity fractures using the long version of the Parents' Postoperative Pain Measure (PPPM), demonstrated that many patients had clinically meaningful pain in the first 48–72 h after ED discharge [[15], [16], [17], [18]].

## 2 Aim of the study

To compare the effectiveness of both types of casting in reducing the pain resulting from DFF using a standardized and validated method of pain assessment. We sought to examine if children who are treated with a long cast after reduction of DFF, experience less pain during the first 48 h after discharge than children who are treated with a short cast.

## 3 Materials and methods

### 3.1 Study design and participants

An open-label, randomized, controlled trial in children undergoing reduction of DFF in the ED, will be conducted, starting in May 2018. Pain after ED discharge will be compared between children treated with long cast immobilization and children treated with short cast immobilization. Parents and patients will be informed regarding the objective of the study.

The study will be conducted in the pediatric ED of Rambam Health Care Campus, in Haifa, Israel. This study was approved by the institutional review board and registered in the Israeli Ministry of Health (MOH) registry (2017-12-20_001956).

### 3.2 Randomization procedure

Randomization will be done in a parallel, stratified fashion using permuted blocks (block size of four) within the following strata: age (5–8 years, 9–12 years) and gender (male, female), to ensure balance between the groups [19].

### 3.3 Patients

Pain after discharge will be assessed in a convenience sample of ED patients aged 4–12 years with a closed fracture of the distal third of the forearm that requires reduction.

#### 3.3.1 Inclusion criteria

A lower age-limit of 4 years was determined, as short casts can slip off the arms of the smallest children. An upper age-limit of 12 years was chosen, as residual deformity is less acceptable in older children because of their diminished remodeling potential [5].

#### 3.3.2 Exclusion criteria (Table 1)

Children with open fractures, Salter–Harris type III or IV fractures, Neurovascular deficit, or known allergy to ibuprofen or acetaminophen will be excluded from the study.

**Table 1:** Inclusion and exclusion criteria.

Inclusion criteria	Exclusion criteria
Age 4–12 years old	Open fractures
Closed fracture of the distal third of the forearm that required reduction	Salter-Harris type-III or IV
	Neurovascular deficit
	Known allergy to ibuprofen or acetaminophen

### 3.4 Trial intervention

Once enrolled, the child's fracture will be managed with closed reduction with the child under deep sedation as per department protocol.

#### 3.4.1 Sedation medications

Sedation medications will be administered according to existing ED protocol, with appropriate doses. Ketamine will be provided in a loading dose of 1 mg/kg, followed by propofol in a loading dose of 1 mg/kg. Additional boluses of 1 mg/kg of propofol and/or ketamine every 1–2 min will be administered as necessary [20].

A senior orthopedic resident will perform the reduction. A below-the-elbow plaster cast will be applied with the use of three-point molding. Once hardened, the cast will be extended above the elbow in patients randomized to the above-the-elbow cast group. Final post reduction radiographs will be taken once the cast is dry [5]. Patients who have unsuccessful reduction will be withdrawn from the study.

#### 3.4.2 Analgesic treatment

After the recovery phase, analgesia will be provided with the use of weight-appropriate doses of medications per ED protocol, using the pain assessment scale according to age (Wong Baker FACES Scale: 4–7 year olds, Visual Analog Scale: 8–12 year olds). Fracture pain can be efficiently treated with ibuprofen, as studies comparing ibuprofen with oral morphine or oxycodone demonstrated that both drugs were associated with clinically significant pain reduction in children who underwent minor orthopedic surgery. Opioids did not provide superior analgesia, but were associated with significantly more adverse effects, making ibuprofen a better analgesic option [[21], [22], [23]].

In the ED, children who report their pain as 1–3 will receive oral acetaminophen 15 mg/kg (maximum of 650 mg). Children who report their pain as 4–6 will receive oral ibuprofen 5 mg/kg. Children who report their pain as 7–10 will receive oral Ibuprofen 10 mg/kg (maximum of 600 mg) [21]. Post reduction and sedation, all children will be observed until full recovery from sedation to discharge. Pain level at discharge from the ED will be less than 5.

At discharge, caregivers of children enrolled in the study will be provided with an information pamphlet and a bottle of ibuprofen syrup to use in case of pain. They will be instructed to use ibuprofen (Nurofen; Reckitt Benckiser Near East, 10 mg/kg, max. 600 mg) every 6 h as needed for pain for 48 h after discharge (max. 8 doses). Parents will be instructed to use acetaminophen 15 mg/kg (maximum of 650 mg) if pain arises between doses of ibuprofen [21]. Parents will be instructed to keep a record of all medications administered to the child and to complete the Parents' Postoperative Pain Measure-Short Form (PPPM-SF, Appendix 1) [[15], [16], [17]]. PPPM-SF forms will be completed by phone 24 h, and 48 h, post discharge. In order to improve parent's compliance with the PPPM-SF, parents will receive the follow-up call from the same study investigator who gave them instruction on how to complete the PPPM-SF [18].

### 3.5 Outcome measures

#### 3.5.1 Primary outcome measure

The primary outcome will be the presence of clinically significant pain during the first 48 h after ED discharge as measured by the PPPM-SF [[15], [16], [17]]. This time frame was selected because children report most significant pain in the first 48 h after discharge [24]. A PPPM-SF score of at least 3 out of 10 signifies clinically significant pain, according to the original study validating the pain scale [15].

#### 3.5.2 Secondary outcome measure

The secondary outcome measure of the study will be the number of ibuprofen and acetaminophen doses administered by parents (total, first day, second day) [24].

### 3.6 Sample size calculation

We performed a power analysis using PPPM-SF as the primary outcome variable, based on a previous study that compared the PPPM-SF and the numeric rating scale in children undergoing ambulatory surgery [17]. In order to detect a between-group median PPPM-SF difference of 2 (20% difference between the groups) with a power of 80% and α of 0.05, our calculation estimates that a sample size of 49 patients per group is needed (98 patients in total). Assuming a drop-out rate of 20%, we calculated a final sample size of 120 patients (60 per group).

### 3.7 Statistical analysis

Patient demographics including age, weight, and gender will be analyzed using the unpaired *t*-test, chi square test, or Fisher's exact test as appropriate. The Mann-Whitney *U* test will be used for comparison of PPPM-SF scores between the groups, and to test differences in the number of doses of ibuprofen and acetaminophen between the groups. A p value less than 0.05 will be considered statistically significant. All statistics will be calculated using the StatsDirect statistical software (v2.6.6, StatsDirect Limited, Cheshire, UK).

## 4 Discussion

Emerging evidence suggests that children who had minor surgery may have clinically meaningful pain in the first 48–72 h after discharge [[15], [16], [17], [18]]. This study will assess post discharge pain in children who had fracture reduction in the ED. We will try to determine if pain is more severe in children who were treated with short cast immobilization compared to children who were treated with long arm immobilization.

This will be the first randomized controlled trial to evaluate post-procedural pain in children who underwent fracture reduction in the ED. We will use a standardized and validated scale that reflects child's self-report of clinically meaningful pain. Importantly, this study is designed to measure not only the overt signs of pain but also behavioral changes associated with pain that may be subtle and potentially missed by parents [18].

The use of the 10-Item PPPM-SF, will encourage a rapid simple assessment of a child's pain. Direct contact with the study investigator will encourage the parents to pay attention to the pain of their children and to use the correct dose of analgesics to treat it [15,18]. We believe that the results of this study will lead to greater awareness and understanding of post-discharge pain management of DDFs for physicians and parents.

## Competing interest

Authors declare that they have no competing interests.

## Funding sources

None declared.

## Conflicts of interest

Non-declared.

## Author's contribution information

MG conceived the idea for the study, extracted the data and carried out the initial analysis, drafted the manuscript, and reviewed the literature; TC assisted in study design, drafted the manuscript, and reviewed the literature; IS designed the study, drafted the manuscript, calculated sample size, and reviewed the literature. IS has full access for the integrity of the data and the accuracy of the analysis.

## Trial registration number

Israel Ministry of Health 2017-12-20_001956.

## Figures and Tables

**Table:** 

*PPSM-SF*			
1	Whine or complain more than usual?	Yes	No
2	Play less than usual?	Yes	No
3	Not do the things s/he normally does?	Yes	No
4	Act more worried than usual?	Yes	No
5	Act more quiet than usual?	Yes	No
6	Have less energy than usual?	Yes	No
7	Eat less than usual?	Yes	No
8	Hold the sore part of his/her body?	Yes	No
9	Groan or moan more than usual?	Yes	No
10	Want to be close to you more than usual?	Yes	No
**Total score**=			

## Appendix 1

### Parents' Postoperative Pain Measure – Short Form (PPPM-SF)

Children sometimes have changes in behavior when recovering from surgery. The following is a list of behaviors that your child may or may not have exhibited while recovering from 'reduction of fracture under sedation'. For each of the behaviors below, circle the appropriate response, yes or no [16].

Parents are asked to complete the measure between a specific time period (i.e., between breakfast and lunch, between lunch and supper, or supper and bedtime). The number of items parents have circled ‘‘Yes’’ are summed for a total score out of 10. A score of at least 3 out of 10 signifies clinically significant pain.

## References

[1] Hughston J.C. (1962). Fractures of the forearm in children. J. Bone Joint Surg. Am..

[2] Reed M.H. (1977). Fractures and dislocations of the extremities in children. J. Trauma.

[3] Landin L.A. (1983). Fracture patterns in children. Analysis of 8682 fractures with special reference to incidence, etiology and secular changes in a Swedish urban population, 1950–1979. Acta Chir. Scand. Suppl..

[4] Worlock P., Stower M. (1986). Fracture patterns in Nottingham children. J. Pediatr. Orthop..

[5] Bohm E.R., Bubbar V., Yong Hing K., Dzus A. (2006). Above and below-the-elbow plaster casts for distal forearm fractures in children. A randomized controlled trial. J. Bone Joint Surg. Am..

[6] Webb G.R., Galpin R.D., Armstrong D.G. (2006). Comparison of short and long arm plaster casts for displaced fractures in the distal third of the forearm in children. J. Bone Joint Surg. Am..

[7] Colaris J.W., Biter L.U., Allema J.H., Bloem R.M., van de Ven C.P., de Vries M.R., Kerver A.J., Reijman M., Verhaar J.A. (2012). Below elbow cast for metaphyseal both-bone fractures of the distal forearm in children: a randomized multicenter study. Injury.

[8] Rahman N., Anwar W., Iqbal M.J., Ahmad I., Khan M.A., Kashif S., Siraj M. (2011). Comparison of below the elbow cast with above the elbow cast in treating distal third forearm fractures in children. J. Surg. Pak. (Int.).

[9] Hendrickx R.P., Campo M.M., van Lieshout A.P., Struijs P.A., van den Bekerom M.P. (2011). Above- or below-elbow casts for distal third forearm fractures in children? A meta-analysis of the literature. Arch. Orthop. Trauma Surg..

[10] van den Bekerom M.P., Hendrickx R.H., Struijs P.A. (2012). Above- or below-elbow casts for distal third forearm fractures in children? An updated meta-analysis of the literature. Arch. Orthop. Trauma Surg..

[11] Vopat M.L., Kane P.M., Christino M.A., Truntzer J., McClure P., Katarincic J., Vopat B.G. (2014). Treatment of diaphyseal forearm fractures in children. Orthop. Rev..

[12] Kumar G. (2006). Comparison of short and long arm plaster casts for displaced fractures in the distal third of the forearm in children. J. Bone Joint Surg. Am..

[13] Pershad J., Monroe K., King W., Bartle S., Hardin E., Zinkan L. (2000). Can clinical parameters predict fractures in acute pediatric wrist injuries?. Acad. Emerg. Med..

[14] Cevik A.A., Gunal I., Manisali M., Yanturali S., Atilla R., Pekdemir M., Gunerli A., Holliman C.J. (2003). Evaluation of physical findings in acute wrist trauma in the emergency department. Ulus. Travma Acil Cerrahi Derg..

[15] von Baeyer C.L., Chambers C.T., Eakins D.M. (2011). Development of a 10-item short form of the parents' postoperative pain measure: the PPPM-SF. J. Pain.

[16] Walther-Larsen S., Aagaard G.B., Friis S.M., Petersen T., Møller-Sonnergaard J., Rømsing J. (2016). Structured intervention for management of pain following day surgery in children. Paediatr. Anaesth..

[17] Brasher C., Gafsous B., Dugue S., Thiollier A., Kinderf J., Nivoche Y., Grace R., Dahmani S. (2014 Apr). Postoperative pain management in children and infants: an update. Paediatr. Drugs.

[18] Thompson R.W., Krauss B., Kim Y.J., Monuteaux M.C., Zerriny S., Lee L.K. (2012). Extremity fracture pain after emergency department reduction and casting: predictors of pain after discharge. Ann. Emerg. Med..

[19] Sealed Envelope Ltd (2017). Create a Blocked Randomisation List. [Online]. https://www.sealedenvelope.com/simple-randomiser/v1/lists.

[20] Ben-Ari M., Chayen G., Steiner I.P., Schinasi D.A., Feldman O., Shavit I. (2018). The effect of in situ simulation training on the performance of tasks related to patient safety during sedation. J. Anesth..

[21] Clark E., Plint A.C., Correll R., Gaboury I., Passi B. (2007). A randomized, controlled trial of acetaminophen, ibuprofen, and codeine for acute pain relief in children with musculoskeletal trauma. Pediatrics.

[22] Poonai N., Bhullar G., Lin K., Papini A., Mainprize D., Howard J., Teefy J., Bale M., Langford C., Lim R., Stitt L., Rieder M.J., Ali S. (2014). Oral administration of morphine versus ibuprofen to manage post fracture pain in children: a randomized trial.

[23] Poonai N., Datoo N.2, Ali S., Cashin M., Drendel A.L., Zhu R., Lepore N., Greff M., Rieder M., Bartley D. (2017). Oral morphine versus ibuprofen administered at home for postoperative orthopedic pain in children: a randomized controlled trial. CMAJ (Can. Med. Assoc. J.).

[24] Unsworth V., Franck L.S., Choonara I. (2007). Parental assessment and management of children's postoperative pain: a randomized clinical trial. J. Child Health Care.

